# Personality Traits and Self-Esteem in Combat and Team Sports

**DOI:** 10.3389/fpsyg.2019.02280

**Published:** 2019-10-09

**Authors:** Željka Bojanić, Jasmina Nedeljković, Dušana Šakan, Petar M. Mitić, Ivana Milovanović, Patrik Drid

**Affiliations:** ^1^Faculty of Law and Business Studies Dr Lazar Vrkatić, University Union Belgrade, Novi Sad, Serbia; ^2^Faculty of Sport and Physical Education, University of Niš, Niš, Serbia; ^3^Faculty of Sport and Physical Education, University of Novi Sad, Novi Sad, Serbia

**Keywords:** neuroticism, extraversion, agreeableness, conscientiousness, openness, combat sports, team sports

## Abstract

The aim of this research was to examine whether psychological variables which make up basic dimensions of personality and self-esteem distinguish competitors in combat sports from competitors in team sports. The research included 149 respondents, aged 19 to 27 years. The Self-Esteem Scale questionnaire was used to measure self-esteem. The BFI inventory was used to measure personality traits according to the Big Five model: Extraversion, Neuroticism, Conscientiousness, Agreeableness, and Openness to Experience. The basic research question is – does the set of psychological variables which make up basic dimensions of personality and self-esteem statistically significantly distinguish competitors in combat sports from the competitors in team sports? Both mean differences and simple discriminant function analyses for competitors in combat/team sports revealed that self-esteem, neuroticism, and conscientiousness were the most important factors distinguishing the two groups. Practical implications, limitations, and future research directions were discussed.

## Introduction

Although the effects of psychology on sports performance have long been recognized, there are more and more psychologists and sports experts dealing with different research domains. Their aim is to take a broader and deeper look at the latent factors of success and failure in sports. Also, one of their aims is to deal with psychological differences between athletes in different sports in order to be able to timely intervene. Besides basic characteristics of personality, self-esteem, as well, is an important feature of athlete competitors.

### Self-Esteem

The notion of self-esteem is determined as a disposition which a person possesses and which represents his/her judgment of his/her own value (Rosenberg, 1965). Coopersmith (1967) defines self-esteem as a set of qualities which an individual observes within himself/herself. According to modern concepts, self-esteem is also defined as respect for one’s own value and importance, as a willingness to be a responsible person and to behave responsibly toward others. Self-esteem appears when a person begins to appreciate and highly value his/her qualities or traits. In other words, self-esteem is the result of evaluating one’s own value, i.e., the outcome of an evaluative orientation toward one’s innermost self; it is the level of belief in one’s own values, the power of belief in one’s own ideas and thoughts, as well as the depth of confidence in one’s own actions (Baumeister, 2013). Self-esteem is not inherent, nor inherited, but it is established, and it changes throughout lifespan under the influence of relationships with others (Baumeister, 2013), especially parents (Qurban et al., 2019).

Rosenberg, whose Self-Esteem Scale was used for the purpose of this research, spoke of self-esteem as being a very important factor in our lives: “Self-esteem directs and activates us in a wide range of activities. It significantly determines our values, our memories and memory processes, our interpretation of events, our evaluation standards and reference points, our goals, our choice of friends, spouse, groups, organizations, professions, and our environment in general. There are few such influential and permeating factors in life as it is self-esteem” (Rosenberg, 1968, p. 345).

The optimum rule applies to self-esteem; when a person has too little of it, he/she functions below his/her potential, and when he/she has too much of it, he/she acquires narcissistic personality traits. High self-esteem is a key factor for success, adequate dealing with failure, and a subjective sense of contentment in life. Persons with high self-esteem take risks more courageously, do not set too high demands on themselves, and highly value themselves. To have high self-esteem means to have a sense of honor and dignity in relation to oneself, one’s own choices, and one’s own life. A person with a high self-esteem has the courage to stand up for himself/herself when he/she is treated below the level he/she believes he/she deserves. One person may be more competent than the other, more experienced, and with higher education, but if the level of his/her self-esteem is lower, he/she is less likely to fight for his/her success. Individuals with high self-esteem deal better with failure compared to those with low self-esteem, feel happier in life, and have lower anxiety (Greenberg et al., 1992).

Previous researches have shown that persons with high self-esteem have better physical and mental health. Persons who have managed to develop self-esteem are more resistant to stress, more confident and do not run away from conflicts, are ready to stand up for themselves, but also to take a critique. Higher self-esteem characterizes persons who are: less prone to depression, more content than most, always endeavor to achieve their goals, see changes as challenges and tend to new experiences, hence sometimes set challenging and demanding goals for themselves (Jordan et al., 2005). Such persons are ambitious and aspire toward constant self-development, both in the emotional, intellectual, and creative fields and in the social and spiritual ones.

More recent findings which use self-esteem as a predictor variable, however, are often contradictory and point to the conclusion that high self-esteem is not at all a guarantee of contentment in life and is associated with a series of problematic behaviors and deviations in cognitive processing. It has, thus, been indicated that high self-esteem is, *inter alia*, also related to self-burdening (taking too much responsibility on oneself), aggressiveness (Kernis et al., 1989), prejudice, discrimination (especially toward those who threaten one’s self-image) (Jordan et al., 2005), narcissism, individual’s tendency to defense reactions, and a number of other behaviors (Baumeister, 2013).

Many authors, as well as self-esteem scales, are based on the idea of self-esteem as a stable trait (Rosenberg, 1965). However, a newer direction of self-esteem research is that high self-esteem can be divided into stable and unstable (fragile) self-esteem. A fragile high self-esteem is when a person shows both, with words and behavior, that he/she has a positive attitude about himself/herself (explicitly), and on an unconscious level he/she has a negative attitude about himself/herself (implicitly). Such persons are prone to destructive and aggressive behavior when their self-image is disturbed (Jordan et al., 2005). Namely, Kernis et al. (2000) have shown that persons with unstable high self-esteem behave as if their self-esteem is always in question, hence they cannot take a critique, have a defensive attitude in interaction with others, show more levels of anger and hostility, and are highly selective in accepting feedback. Furthermore, they brag about their successes to their friends, while in doing so using self-magnification and being more self-burdened (Kernis et al., 2000). The feeling of one’s own self-esteem in mature years is also based on respect for others. Persons with high self-esteem do not perceive other people as threats, nor do they think ahead that they will be rejected, humiliated, deceived, or betrayed by others because they are aware of themselves and their qualities. Thus, they treat others with respect, justness, and good intentions.

High self-esteem is desirable, especially in individualistic cultures, because it is a predictor of success in various spheres of life, but some social psychologists have dealt with the research of negative sides of high self-esteem. One of the more significant studies relates to the connection between self-esteem and anger and hostility (Kernis et al., 1989). A group of scientists (Kernis et al., 1989) compared the level and stability of self-esteem with the amount of anger and hostility which individuals experience. Self-esteem stability was tested by multiple global self-esteem tests in natural conditions. The researchers have found that the predictor of anger and hostility is not only the level of self-esteem but also its stability. High but unstable self-esteem is in a statistically significant correlation with the extremely high tendency of experiencing anger and enmity, or hostility.

Persons with a lack of self-esteem have doubts about their abilities and they get up courage to take risks only when they are certain that they are fully competent and that they meet all the conditions. Furthermore, researches have shown that low self-esteem leads to certain negative occurrences, such as: delinquent behavior, depression, bulimia, tendency for mental illnesses, and dissatisfaction with relationships (Baumeister et al., 2005; Bulik et al., 2007).

Baker and McNulty (2013) state that persons with low self-esteem prefer safety and familiar situations, avoid challenging goals and, thus, reflect low self-esteem. They are not direct in communication, they often fear to openly say what they think and feel, first and foremost because they themselves are uncertain in their thoughts, and then because they fear the reactions of others. Such persons cannot stand failures well. Every time they do not achieve their goal, to them, that is another proof that they are worthless and unsuccessful. Trapped in their thinking and self-evaluation scheme, they usually do not even consider other reasons for failure. Persons with lower self-esteem often think that other people do not have a high opinion of them, as well, which makes them feel rejected and they rarely decide to initiate social contacts. A fewer number of social contacts leads to fewer opportunities for creating deeper interpersonal relationships, from which a person can expect social support. Thus, low self-esteem affects the size and the quality of a person’s social network.

### Personality Traits

The Big Five model describes five dimensions of personality: Extraversion, Agreeableness, Conscientiousness, Neuroticism, and Openness to experience. Extroversion implies that individuals are sociable, while introversion implies that they are quiet and reserved (John et al., 2008). Extraversion is characterized by openness, assertiveness, and a high level of energy (John et al., 1991). Persons with a high score on extraversion are more open, more persistent, more talkative, and more social than those with a low score on extraversion, who are shy, quiet, and aloof (Larsen and Buss, 2009). Extraversion is associated with values of achievement and hedonism (Roccas et al., 2002), but also with goals relating to an exciting lifestyle (Roberts et al., 2004).

Agreeableness means that persons are cooperative and kind, and not rough (John et al., 2008). This dimension is characterized by benevolence and trust. It can be seen as a combination of friendship and harmonization (John et al., 1991). Persons with a high score in this dimension are warm, sympathetic, and honest, while persons with a low score in this dimension are unkind, often rude, and sometimes even cruel (Larsen and Buss, 2009). Agreeableness is associated with harmonious family relationships, good partnership relationships (Roberts et al., 2004), but also with prosocial values (Haslam et al., 2009).

Conscientiousness is characterized by orderliness, responsibility, and reliability; hence this trait is sometimes called reliability, as well (John et al., 1991). Conscientious persons are hardworking, disciplined, pedantic, and they devote a lot of time to organization. These are persons who are intrinsically motivated and who make a lot of effort to be successful in what they do (Larsen and Buss, 2009). Conscientiousness is associated with goals of achievement (Costa and McCrae, 1988), but also with goals relating to interpersonal relationships (Roberts et al., 2004). Therefore, it can be said that conscientious persons are oriented toward goals, the execution of a task, and that they are reliable and punctual thereby (Larsen and Buss, 2009).

Neuroticism is characterized by uneasiness and a polar opposite of emotional stability (John et al., 1991) and such individuals are prone to experiencing anxiety, depression, and irritation (John et al., 2008). Persons with a high score on neuroticism are insecure, often have mood swings, while emotionally stable people are calmer, more relaxed, and more stable (Larsen and Buss, 2009). Also, a high score on neuroticism suggests suggestibility or susceptibility to suggestion, lack of persistence against obstacles, dragginess, and poor fluency, or the existence of rigidity. Also, characteristics of neuroticism include the feeling of inferiority, nervousness, avoidance and intolerance of effort, dissatisfaction, sensitivity, irritability, and touchiness. On the other hand, emotional stability is associated with strategy, i.e., the way in which a person overcomes stress and various obstacles in life (Larsen and Buss, 2009). Emotionally stable persons do not get disturbed, except when the issues in question are very strong stressors for them personally. Emotionally stable persons can experience neurosis symptoms only when in a situation of long-term and strong stress (Larsen and Buss, 2009).

Openness to experience is characterized by originality, curiosity, and ingenuity. This factor is sometimes called culture because of its emphasis on intellect and independence (John et al., 1991). Individuals open to experience have broad interests and a fine taste for art and beauty (John et al., 2008). Persons with a high score in this dimension are creative, imaginative, and since they have a broad range of interests, like to explore the unknown, while persons with low scores in this dimension are of conventional appearance and behaviors, narrowed interests, prone to conservative attitudes, and tend to prefer what is already known in relation to the unknown (Larsen and Buss, 2009). Openness to experience is often associated with autonomy (Roccas et al., 2002).

### Personality Traits and Self-Esteem in Athletes

Although, according to Allen et al. (2013), psychological researches of athletes’ personality are in a certain crisis in the field of sports psychology, there is a vast body of research with findings on identifying differences between personality traits of athletes in different sports (Peterson et al., 1967; Dowd and Innes, 1981; Geron et al., 1986; Malinauskas et al., 2014; Olmedilla et al., 2019). These researches showed that athletes participating in team sports have higher levels of extraversion in comparison to athletes participating in individual sports. Then, athletes participating in team sports have lower level of conscientiousness than athletes participating in individual sports (Nia and Besharat, 2010; Allen et al., 2011; Malinauskas et al., 2014). Also, athletes participating in high-risk sports have higher levels of extraversion and lower levels of conscientiousness than athletes participating in low-risk sports (Castanier et al., 2010). Results of meta-analysis showed that high-risk sport participants had higher levels of sensation seeking, extraversion and impulsivity and lower levels of neuroticism, sensitivity to punishment in comparison to individuals who do not undertake such activities (McEwan et al., 2019). When comparing athletes with non-athletes, findings indicate higher levels of extraversion and higher levels of emotional stability in population of athletes than in non-athletes (Egan and Stelmack, 2003; McKelvie et al., 2003), as well as higher openness to experience (Hughes et al., 2003). Further, it was shown that karate masters have low neuroticism and high conscientiousness, depending on their level of mastery, so that more experienced athletes have more stable personality than those less experienced (Piepiora et al., 2018).

In one study on Tunisian athletes (Ali et al., 2013), the results revealed that self-esteem was higher in a group of athletes participating in individual sports than in athletes participating in team sports, while aggressiveness was higher in team sports than in individual sports. When comparing levels of aggression in individual non-aggressive sports like tennis, dancing, swimming with individual combat sports, results reveal that non-aggressive sports entail higher levels of self-esteem and verbal aggression, hostility, and anger (Ali et al., 2013). Other studies indicated contrary results, claiming higher levels of aggression in combat sports than in other individual sports (Zillmann et al., 1974; Bredemeier et al., 1987). Even though some studies show no difference in self-esteem between individual and team athletes (Akelaitis and Malinauskas, 2018), higher levels of self-esteem are important salient of better coping strategies in sport stress management (Káplanová, 2018). According to a literature review which indicated mixed findings in this field, we have tested differences in personality traits and self-esteem in athletes participating in two groups of sports, in team sports and in combat sports.

### Research Question

Does the set (framework, profile) of psychological variables which make up basic dimensions of personality and self-esteem statistically significantly distinguish competitors in combat sports from the competitors in team sports?

## Methods

### Participants

One hundred and forty nine undergraduate students of all years of the Faculty of Sport and Physical Education at the Universities of Novi Sad and Niš (*N* = 149) were surveyed. The sample consists of 87 (58.4%) males and 62 (41.6%) females of the average age of 20.95 years (*SD* = 1.787). The surveyed students are at the same time competitors in sports which they have trained on average 10.73 years (*SD* = 3.93). Competitors in combat sports (27.5% of the total sample) train wrestling, karate, judo, jiu-jitsu, kick boxing, MMA, and taekwondo. Competitors in team sports (72.5% of the total sample) train football, basketball, volleyball, and handball.

### Instruments

The Self-Esteem Scale questionnaire was used to measure self-esteem (SES, Rosenberg, 1965). The Scale has 10 items and the reliability on our sample is α = 0.85. The BFI inventory (John and Srivastava, 1999) has 44 items and it was used to measure personality traits according to the Big Five model: Extraversion (α = 0.78), Neuroticism (α = 0.70), Conscientiousness (α = 0.76), Agreeableness (α = 0.76), and Openness to Experience (α = 0.73).

### Procedure

The research was carried out in year 2018, with previously obtained approvals from the ethical committees of the faculties and voluntary consents of the students. The testing was conducted in a paper-and-pencil test form and it lasted for 30 min.

### Data Analysis

In the data analysis, descriptive measures, mean differences, and canonical discriminant analysis were used.

## Results

Since univariate tests do not take into account the relationship among variables, a discriminant function analysis was also performed on the six variables. The values of group centroids (mean discriminant scores for each group) for competitors in combat and team sports were −0.57 and 0.216, respectively. The discriminant function obtained for the six factors, Wilks λ = 0.889, Chi-square (6) = 16.94, *p* < 0.01. Refer to Table 1 for the entry order of the six variables and their corresponding standardized discriminant function coefficients.

**TABLE 1:** Means, standard deviations, *t*-tests, standardized discriminant function coefficients, and correlations between discriminant scores and variable raw scores for competitors in combat and team sports.

	**Sports**	**Mean**	***SD***	***F***	**Stand. Can. Disc. Fun. Coeff.**	**Corr. Disc. Var. Raw**
Self-esteem	Combat	23.38	1.705	4.518^∗^	0.617	0.496
	Team	24.10	1.874			
Neuroticism	Combat	25.05	3.507	4.109^∗^	0.729	0.473
	Team	26.28	3.696			
Extraversion	Combat	26.90	2.976	0.100	–0.137	–0.074
	Team	26.59	3.304			
Agreeableness	Combat	31.72	3.404	1.684	–0.229	–0.303
	Team	31.00	3.373			
Conscientiousness	Combat	32.32	3.284	5.649^∗^	–0.562	–0.555
	Team	30.63	4.194			
Openness	Combat	36.87	6.389	0.864	0.198	–0.217
	Team	35.80	6.085			

*^∗^Differences are significant at the 0.05 level; Stand. Can. Disc. Fun. Coeff., Standardized Discriminant Function Coefficients; Corr. Disc. Var. Raw, Correlations Between Discriminant Scores and Variable Raw Scores For competitors in combat and team sports.*

The obtained canonical discriminant function is: Discriminant score = 0.729^∗^ Neuroticism + 0.617^∗^ Self-esteem + 0.198^∗^ Openness – 0.562^∗^ Conscientiousness – 0.229^∗^ Agreeableness – 0.137^∗^ Extraversion.

**FIGURE 1:**
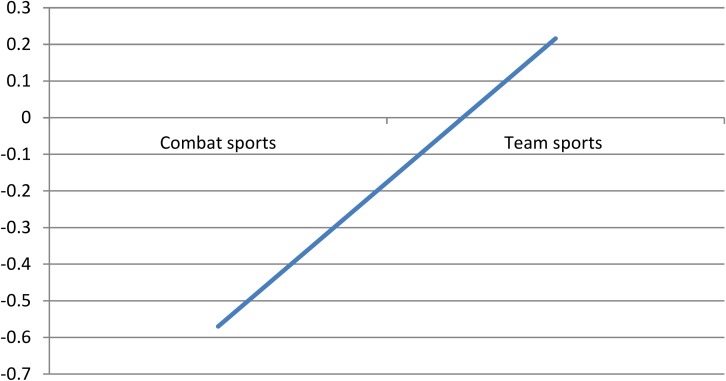
Group centroids – distance between groups.

According to the group centroids it can be said that competitors in combat sports are characterized by a positive high score in conscientiousness, positive scores in agreeableness and extraversion (Figure 1). They are also characterized by low neuroticism and self-esteem, and low scores in openness to new experiences. Competitors in team sports have the opposite traits.

## Discussion

Main objective of our research was to examine individual differences in personality traits and self-esteem, as important psychological predictors of optimal adjustment and sports performance, in athletes participating in team sports and athletes participating in combat sports.

The largest group difference was obtained in the dimension of Conscientiousness. Competitors in combat sports are more conscientious than competitors in team sports. This result suggests that athletes participating in combat sports, because of the very nature of these sports, rely on their own strengths and confidence in personal skills and knowledge. In addition, combat sports are, apart from the competitive aspect, also envisaged for the development of self-control, persistence, and self-discipline (Bernacka et al., 2016; Kostorz et al., 2017), which make up facets of the Conscientiousness trait (Costa and McCrae, 1988; John et al., 1991; Larsen and Buss, 2009).

Second result suggests that competitors in team sports have significantly higher self-esteem than competitors in combat sports. This result is inconsistent with previous studies (Ali et al., 2013) which indicate that self-esteem is higher in team than in individual sports or those that show no differences at all (Akelaitis and Malinauskas, 2018). However, in our study, individual sports are combat sports, while in the study of Ali et al. (2013) these sports are mixed with other non-aggressive sports such as tennis and the like. Our assumption is that, given that self-esteem depends on social influences (Baumeister, 2013; Qurban et al., 2019), it is more fragile in individual sports in which there is no support from the teammates. Further, this finding can be related to body image of athletes. Although sport and physical activity in general are associated with more positive body image (Hausenblas and Downs, 2001; Sabiston et al., 2019), there are not relevant findings about the relations of body image and concrete sport. So, we could assume that lower self-esteem in combat athletes in our research is due to a specific appearance of fighters. Another assumption could be that lower-esteem in a subsample of combat athletes is due to the period of adolescence, in which our respondents currently are, that often corresponds with lower aspects of the identity status. Other than that, our findings can be explained in the context of organizational stressors that can impact athletes’ self-esteem, such as coaching stressors and competition demands (Tamminen et al., 2019), but this assumption needs more thorough further examination.

Neuroticism is significantly higher among competitors in team sports compared to competitors in combat sports. This result is based on the fact that in addition to mastering basic defense skills, the aim of training combat sports is to work on focus, the feeling of peace – deep connection with one self and work on self-control – control of impulses, because in combat everything depends on themselves, there is no division of responsibilities as in team sports (Allen et al., 2013; Bernacka et al., 2016; Kostorz et al., 2017). Another possible way to explain our result is that team sport athletes often experience negative interpersonal encounters with their teammates regarding team roles, communication difficulties or even selfishness (Rainey and Petkari, 2019) that may trigger manifestation of their higher levels of Neuroticism.

Our results do not indicate statistically significant differences in Extraversion which were registered in other studies (Nia and Besharat, 2010; Allen et al., 2011), nor in Openness to experience and Agreeableness, which were also not registered in other samples.

## Conclusion

Our study has, to some extent, contributed to the knowledge of differences in individual differences between athletes in team sports and athletes in combat sports. The findings can be useful in the training process and in the psychological preparation of athletes. More precisely, the results show which personality aspects should be accentuated when working with athletes in martial arts (for example, self-respect) and which with the athletes in collective sports (for example conscientiousness). The limitations of the study are the unequal number of athletes in the two examined groups, as well as the potentially insufficient number of included variables which could in subsequent studies explain the obtained differences – such as the issue of identity status, physical self-awareness, and motivation for doing sports. The limitation relating to the number of examinees can be overcome by including not only a larger number of athletes but also athletes from various martial arts and collective sports.

## Data Availability Statement

The datasets analyzed in this manuscript are not publicly available. Requests to access the datasets should be directed to ŽB, zeljka.bojanic@vojvodina.gov.rs.

## Ethics Statement

All subjects gave written informed consent in accordance with the Declaration of Helsinki. The protocol was approved by the local ethics committee of the Faculty of Sport and Physical Education, University of Novi Sad.

## Author Contributions

ŽB, JN, DŠ, PM, IM, and PD designed the experiments, analyzed and interpreted the data, edited the manuscript, and approved the final version to be published and are accountable for all aspects of the work. ŽB and PD performed the experiments and wrote the initial draft of the manuscript.

## Conflict of Interest

The authors declare that the research was conducted in the absence of any commercial or financial relationships that could be construed as a potential conflict of interest.
